# APOBEC3-mediated restriction of RNA virus replication

**DOI:** 10.1038/s41598-018-24448-2

**Published:** 2018-04-13

**Authors:** Aleksandra Milewska, Eveline Kindler, Philip Vkovski, Slawomir Zeglen, Marek Ochman, Volker Thiel, Zenon Rajfur, Krzysztof Pyrc

**Affiliations:** 1 Microbiology Department, Faculty of Biochemistry, Biophysics and Biotechnology, Jagiellonian University, Gronostajowa 7, 30-387 Krakow, Poland; 2 Virogenetics Laboratory of Virology, Malopolska Centre of Biotechnology, Jagiellonian University, Gronostajowa 7a, 30–387 Krakow, Poland; 3 Institute for Virology and Immunology, Bern and Mittelhäusern, Bern, Switzerland; 4 Department of Infectious Diseases and Pathobiology, Vetsuisse Faculty, University of Bern, Länggassstrasse 122, Bern, Switzerland; 5 Graduate School for Cellular and Biomedical Sciences, University of Bern, Bern, Switzerland; 6 Department of Cardiac Surgery and Transplantology, Silesian Center for Heart Diseases, Marii Curie-Skłodowskiej 9, 41-800 Zabrze, Poland; 7 Head of Histology Department, Medical Department, University of Opole, Opole, Poland; 8 Department of Pharmacology, School of Medicine with the Division of Dentistry in Zabrze, Medical University of Silesia in Katowice, Katowice, Poland; 9 Institute of Physics, Faculty of Physics, Astronomy and Applied Computer Sciences, Jagiellonian University, Lojasiewicza 11, 30-348 Krakow, Poland

## Abstract

APOBEC3 family members are cytidine deaminases with roles in intrinsic responses to infection by retroviruses and retrotransposons, and in the control of other DNA viruses, such as herpesviruses, parvoviruses and hepatitis B virus. Although effects of APOBEC3 members on viral DNA have been demonstrated, it is not known whether they edit RNA genomes through cytidine deamination. Here, we investigated APOBEC3-mediated restriction of *Coronaviridae*. In experiments *in vitro*, three human APOBEC3 proteins (A3C, A3F and A3H) inhibited HCoV-NL63 infection and limited production of progeny virus, but did not cause hypermutation of the coronaviral genome. APOBEC3-mediated restriction was partially dependent on enzyme activity, and was reduced by the use of enzymatically inactive APOBEC3. Moreover, APOBEC3 proteins bound to the coronaviral nucleoprotein, and this interaction also affected viral replication. Although the precise molecular mechanism of deaminase-dependent inhibition of coronavirus replication remains elusive, our results further our understanding of APOBEC-mediated restriction of RNA virus infections.

## Introduction

Apolipoprotein B mRNA-editing enzyme, catalytic polypeptide-like type 3 (APOBEC3) proteins are Zn^2+^ - dependent cytidine deaminases that belong to the APOBEC superfamily. APOBEC3 enzymes are considered to be part of the intrinsic defense system in eukaryotic cells, and can inhibit replication of some viruses and restrict mobile genetic elements. All APOBEC3 proteins share a cytidine-deaminase domain, which is identifiable by the primary amino acid motif His-Xaa-Glu-Xaa_23–28_-Pro-Cys-Xaa_2–4_-Cys, in which Zn^2+^ is coordinated. Deamination of cytidine results in its conversion to uridine. During this process, the ammonium group on C_4_ of cytidine undergoes nucleophilic attack by an activated water molecule and the APOBEC3 glutamate residue.

A single copy of a gene encoding APOBEC3 is found in the genomes of rodents, cats, pigs and sheeps, with two genes in the genomes of cows, three the genomes of dogs and horses and seven genes in the human genome, encoding A3A, A3B, A3C, A3D, A3F, A3G and A3H^1^. The mechanisms and selective pressures that led to the APOBEC3 gene expansion in humans are unclear, but it is believed that all the genes originated from duplication of a single-copy primordial APOBEC3 gene. This multiplication may have provided protection from genomic instability caused by human endogenous retroviruses and retrotransposons^2^.

The best-studied protein within the human APOBEC3 family is A3G. Its function as an antiviral factor was discovered in 2002 through cDNA-transfer experiments that were designed to identify a cellular suppressor of an HIV-1 accessory protein, the virion infectivity factor (Vif). The primary antiretroviral activity of A3G requires its encapsidation into HIV-1 particles, and involves cytidine deamination of the reverse-transcribed first viral DNA strand, with mutations becoming fixed as G to A changes upon second-strand synthesis. Mutational frequencies can exceed 10% of all G residues (hypermutation), and may result in inhibition of viral replication. This activity is blocked by Vif protein, which specifically binds to and ubiquitinates A3G, directing it for degradation in the proteasome^1,3,4^. Other APOBEC3 proteins have been found to affect a wide variety of viruses, including other retroviruses (e.g., simian immunodeficiency virus (SIV), equine infectious anemia virus (EIAV), murine leukemia virus (MLV), foamy viruses), and DNA viruses (e.g., hepatitis B virus, parvovirus)^5–14^. As well as the inhibition of viral replication by hypermutation, APOBEC3-induced mutations also cause degradation of viral genomic material, mediated by cellular DNA-repair mechanisms^15^.

The RNA-editing capability of APOBEC3 proteins was identified in 2004^16^, since when extensive mutational activity of A3A on RNA transcripts in monocytes and macrophages has been demonstrated^17^. Our current understanding of the APOBEC3 proteins suggests that they may also constitute an innate defense system against RNA viruses. For example, A3G known to be involved in restriction of three RNA pathogens (the mumps, measles and respiratory syncytial viruses), but whether this effect is associated with cytidine deamination is not yet known^18^.

Members of the family *Coronaviridae* are positive-strand RNA viruses with large genomes, ranging in size from 27 to 32 kb. Six human coronaviruses (HCoVs) have been identified, four of which (HCoV-OC43, HCoV-229E, HCoV-NL63 and HCoV-HKU1) circulate continuously in human populations. These four viruses cause the common cold in otherwise healthy adults, and may cause more severe symptoms in young, elderly and immunocompromised individuals^19–22^. Other coronaviruses that infect humans can cause severe and life-threatening diseases. In 2002, severe acute respiratory syndrome coronavirus (SARS-CoV) emerged and affected ~8,000 individuals, causing ~800 deaths^23,24^. Middle East respiratory syndrome coronavirus (MERS-CoV) is the most recently identified coronaviruse that infects humans. MERS-CoV was isolated in 2012 and is associated with severe pneumonia and renal failure, with a mortality rate of ~30%^25,26^.

HCoV-NL63 was identified in 2004 and is distributed worldwide, with highest prevalence during winter and early spring in temperate climate^27^. HCoV-NL63 accounts for a considerable number of hospitalisations among children <18 years of age, the elderly and immunocompromised individuals^19^. A notable characteristics of coronaviruses is that their genomes are highly U/A-rich and C/G-poor. For example, HCoV-NL63 has 39% U and 27% A content, with only 14% C and 20%, G nucleotides^28^. The explanations for this bias is not yet known^28,29^. One possibility is that, over an evolutionary timescale, genomes of coronaviruses might have been shaped by cytidine deamination. Our aim, therefore, was to determine whether APOBEC3 proteins might be responsible for this modification. Our results demonstrate that three of the APOBEC3 proteins (A3C, A3F and A3H) can inhibit coronaviral infection *in vitro*.

## Results

### APOBEC3-family gene expression is upregulated in HCoV-NL63-infected cells

**Figure 1:**
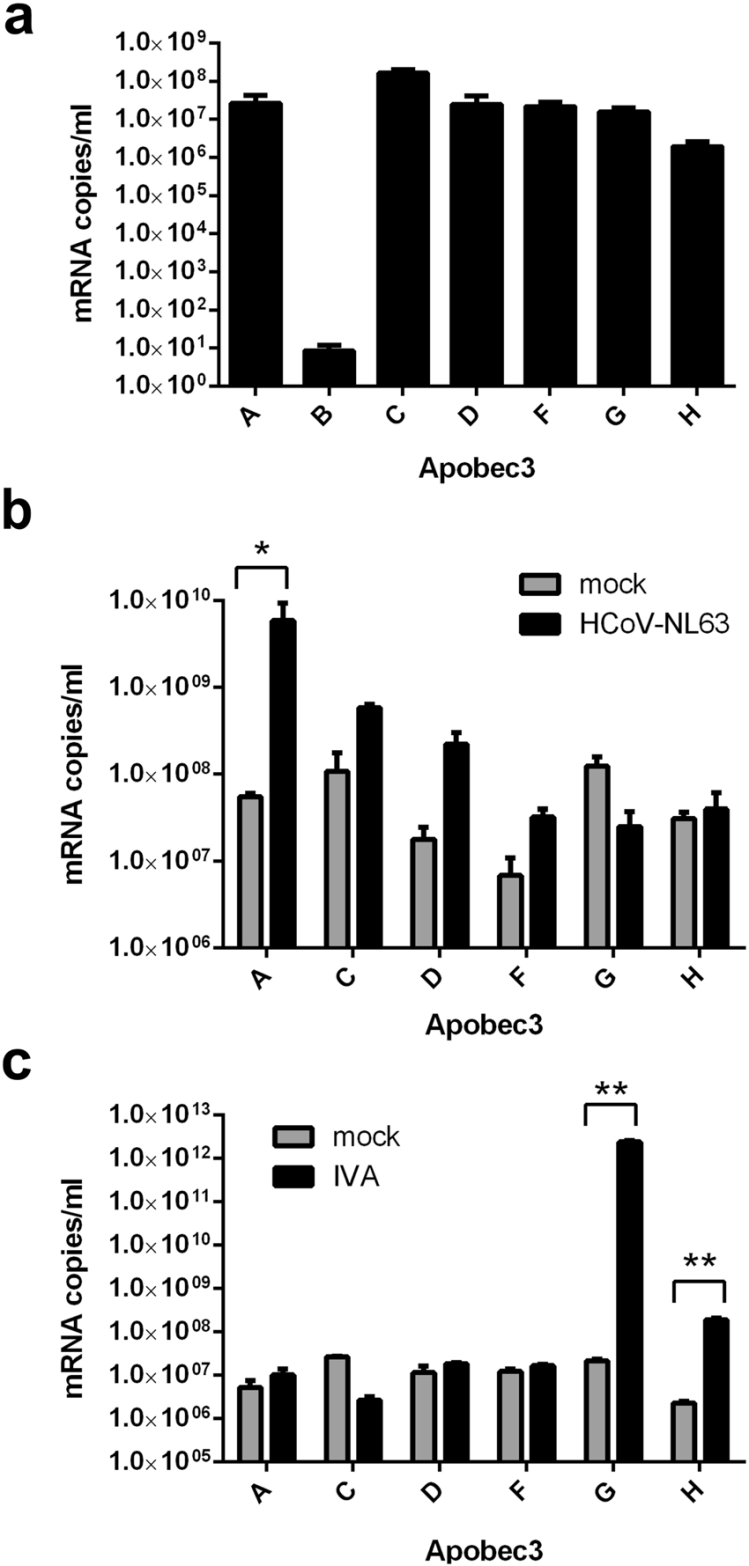
APOBEC3 gene expression in human airway epithelium (HAE) cell culture. APOBEC3 gene expression was analysed in 3D HAE cultures. mRNA levels were evaluated by quantitative RT-PCR. Expression was assessed (**a**) in uninfected HAE cultures, (**b**) in cultures infected with human coronavirus (HCoV)-NL63 and (**c**) in cultures infected with influenza A virus (IVA) strain H3N2. Results are presented as mRNA copy number per ml. Infection was performed at 400 TCID_50_ per ml, for 4 days at 32 °C (for HCoV-NL63) or for 2 days at 37 °C (for IVA). Each experiment was performed at least three times with clinical material from different donors, and with two technical replicates. For comparisons by Student’s *t*-test, *Indicates *P* < 0.05; **Indicates *P* < 0.005.

Modulation of human APOBEC3-family transcript levels in human airway epithelium (HAE) cultures during coronavirus infection was evaluated by quantitative RT-PCR (qRT-PCR) with primers and standards developed in our lab. The level of each APOBEC3 mRNA was analysed in fully differentiated cells from healthy adults, and a high level of expression was observed for six out of seven human APOBEC3 genes (Fig. 1a). The same six genes (*APOBEC3A*, *APOBEC3C*, *APOBEC3D*, *APOBEC3F*, *APOBEC3G* and *APOBEC3H*) were expressed during HCoV-NL63 infection, and these genes were analysed in subsequent experiments.

HAE cultures were infected with HCoV-NL63 and cultured for 5 days at 32 °C. As a control, HAE cultures were infected with influenza A H_3_N_2_ virus, which induces considerable upregulation of *APOBEC3G*^30^. RT-qPCR analysis of total RNA extracted from infected cells revealed upregulation of *APOBEC3A*, *APOBEC3C*, *APOBEC3D* and *APOBEC3F* transcripts in HCoV-NL63-infected cells relative to mock-infected cells, with an almost 100-fold increase in *APOBEC3A* expression (Fig. 1b). As anticipated, *APOBEC3G* was upregulated in the influenza-infected cells, and *APOBEC3H* expression also increased (Fig. 1c).

### Expression of APOBEC3 proteins in cultured cells

**Figure 2:**
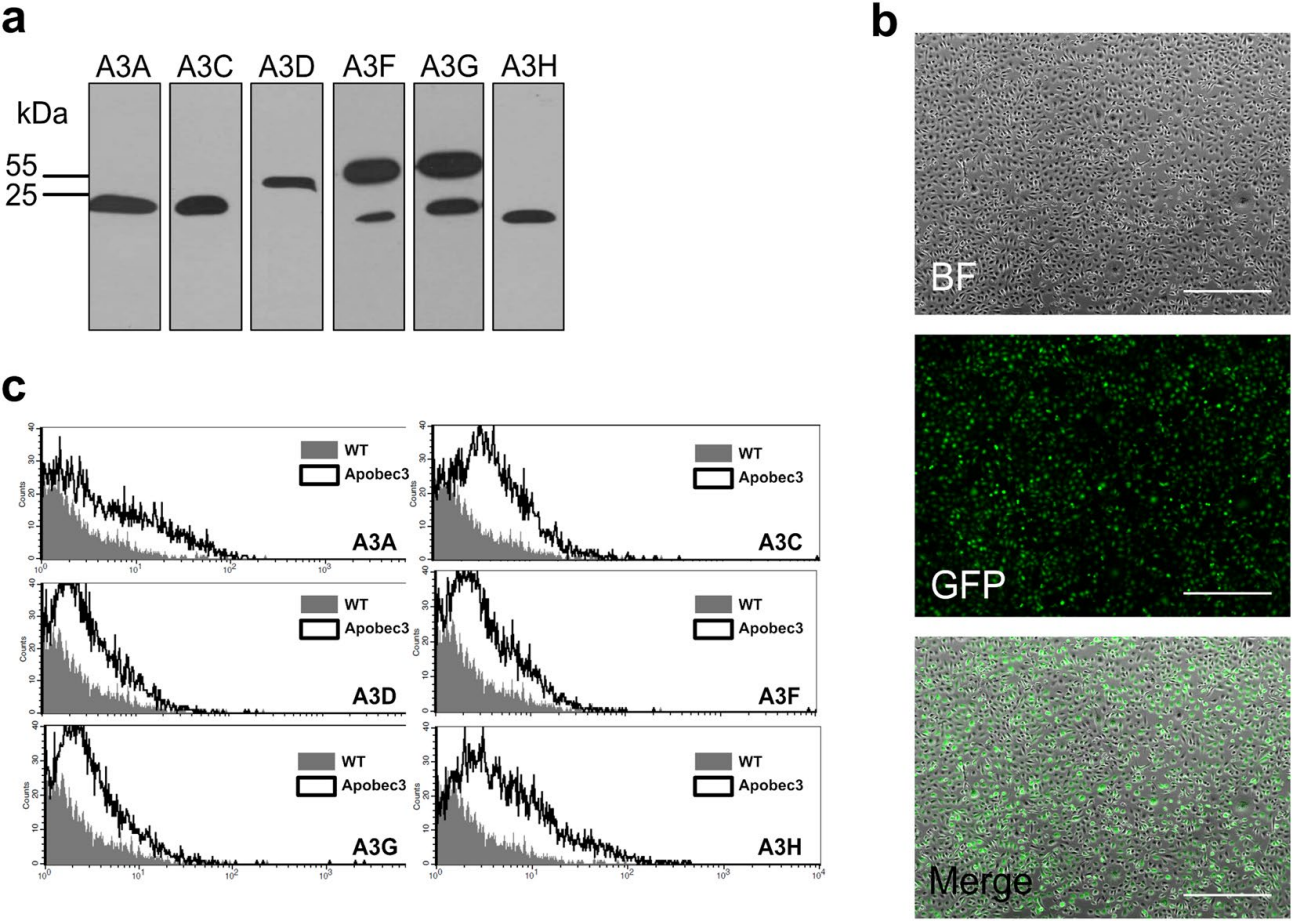
HA-tagged APOBEC3 protein expression after DNA or mRNA transfection. (**a**) 293T cells were transiently transfected with plasmid DNA encoding individual APOBEC3 proteins with a C-terminal HA tag. APOBEC3 proteins were detected by western blotting using HA-specific antibodies. (**b**) LLC-Mk2 cells were transfected with capped and polyadenylated green fluorescent protein (GFP) mRNA and cultured for 24 h at 37 °C in an atmosphere containing 5% CO_2_. Green fluorescence was visualised with a fluorescence microscope. BF = bright field. Scale bar = 650 µm. (**c**) LLC-Mk2 cells were transfected with capped and polyadenylated mRNA encoding individual APOBEC3 proteins and cultured for 48 h at 37 °C in an atmosphere containing 5% CO_2_. Protein expression was analysed with flow cytometry using HA-specific antibodies.

Plasmids encoding individual APOBEC3 proteins were prepared and transfected into 293T cells, which were cultured and analysed for protein expression by western blotting (Fig. 2a, Supplementary Figure 1). For expression in the naturally permissive LLC-Mk2 cell line, in which the DNA transfection efficiency is very low (data not shown), mRNA transcripts encoding APOBEC3 proteins or green fluorescent protein (GFP) were prepared by *in vitro* transcription. Transfection of LLC-Mk2 cells was efficient, reaching ~80% for the control GFP mRNA (Fig. 2b). Expression of HA-tagged APOBEC3 proteins in mRNA transfected LLC-Mk2 cells, measured by flow cytometry, was similar for all proteins (Fig. 2c).

### Human APOBEC3 proteins A3C, A3F and A3H inhibit HCoV-NL63 replication

**Figure 3:**
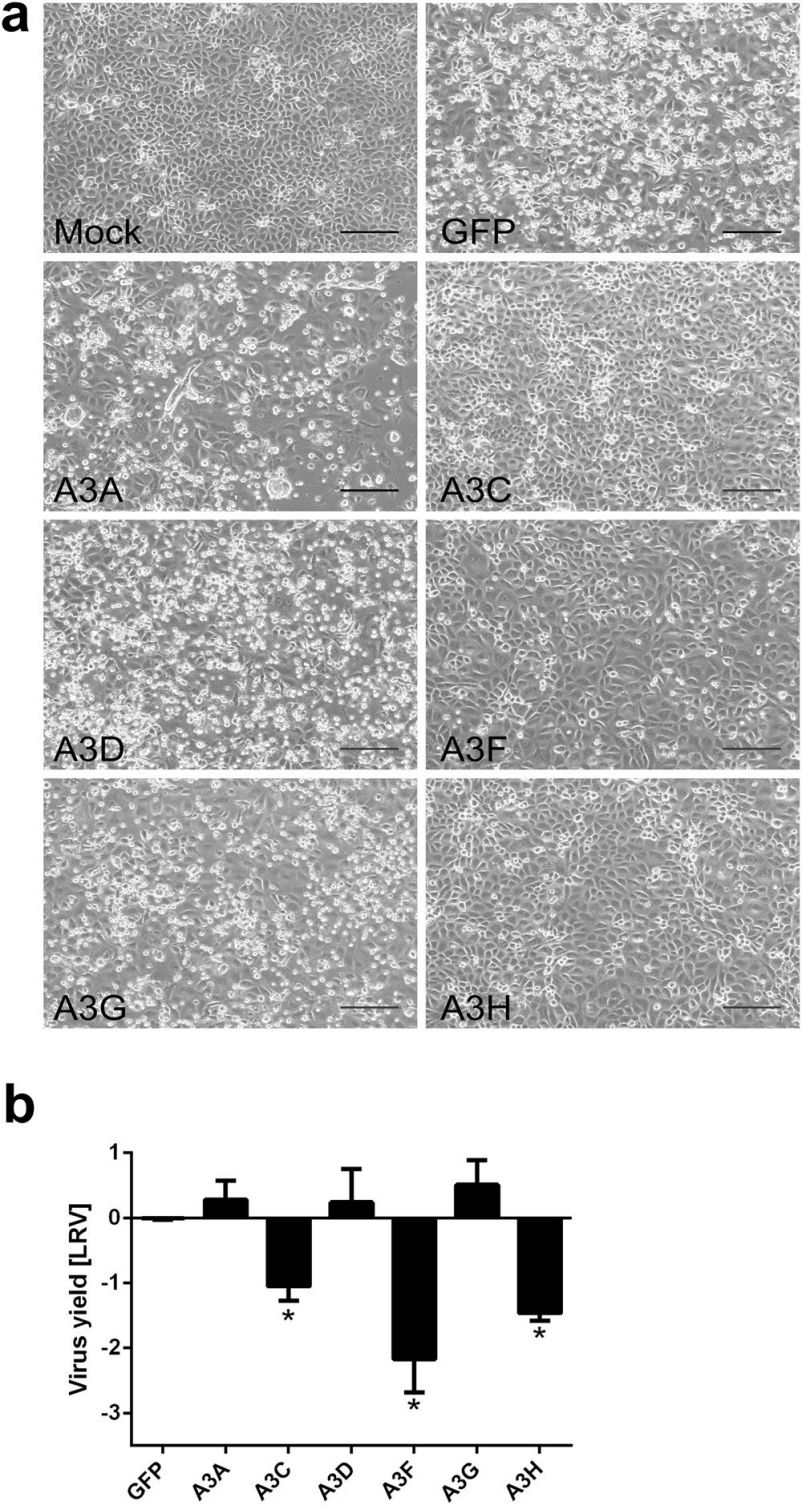
Human coronavirus (HCoV)-NL63 infection is inhibited in LLC-Mk2 cells expressing the individual APOBEC3 proteins A3C, A3F and A3H. (**a**) LLC-Mk2 cells were transfected with capped and polyadenylated mRNAs encoding individual APOBEC3 proteins. Following 24 h incubation at 37 °C in an atmosphere containing 5% CO_2_, cells were infected with HCoV-NL63 at 400 TCID_50_ per ml. After a further 120 h incubation at 32 °C in an atmosphere containing 5% CO_2_, development of cytopathic effect (CPE) was assessed by observation with an inverted microscope. Scale bar = 170 µm. (**b**) Cell-culture supernatant was harvested at 120 h post-infection, and viral RNA was isolated and reverse transcribed. Virus yield was determined by quantitative RT-PCR, and is presented as Log Reduction Value (LRV). Each experiment was performed at least three times, with two technical replicates. For comparisons by Student’s *t*-test, *Indicates *P* < 0.05.

mRNA-transfected LLC-Mk2 cells were infected with HCoV-NL63 and cultured for 5 days at 32 °C. No cytopathic effect (CPE) was observed in cells expressing A3C, A3F and A3H proteins, but CPE was observed in untransfected or GFP-transfected cells, and in cells expressing A3A, A3DE and A3G (Fig. 3a). Analysis of HCoV-NL63 replication showed a 1.5–2 log reduction in virus yield in cells expressing A3C, A3F and A3H, relative to the control sample. By contrast, HCoV-NL63 replication was not inhibited in cells expressing GFP, A3A, A3D or A3G (Fig. 3b).

### APOBEC3-mediated HCoV-NL63 restriction is associated with cytidine deamination

**Figure 4:**
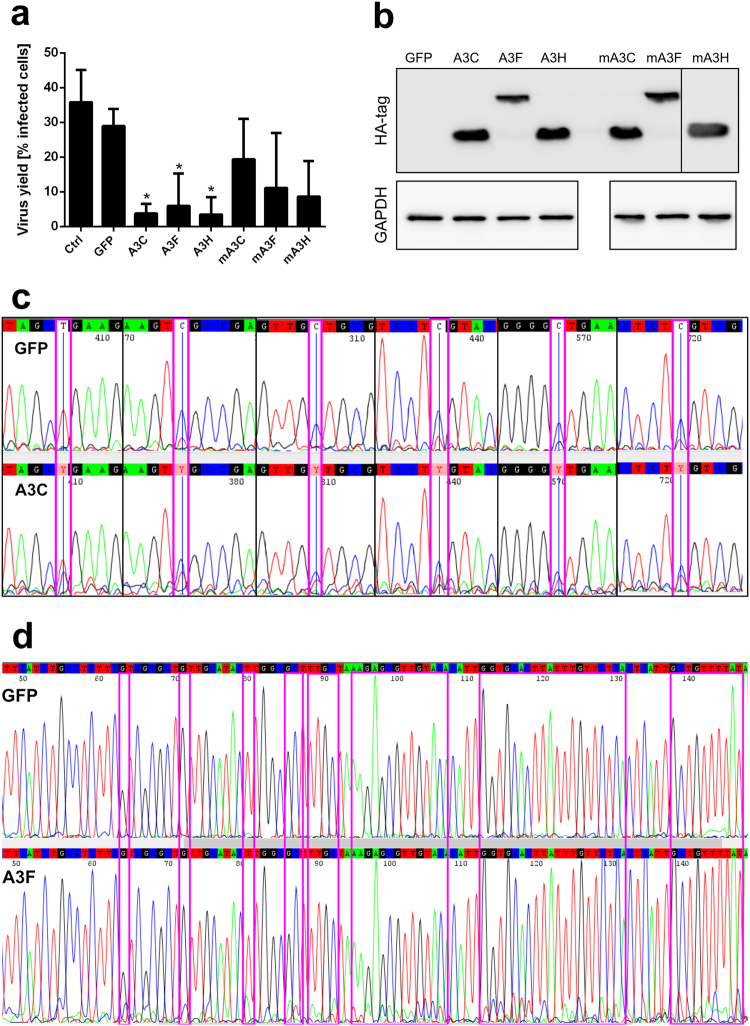
Cytidine deamination is relevant for HCoV-NL63 inhibition. (**a**) LLC-Mk2 cells were transfected with capped and polyadenylated mRNA encoding active or catalytically inactive (m) APOBEC3 proteins. Following incubation for 24 h at 37 °C in an atmosphere containing 5% CO_2_, cells were infected with human coronavirus (HCoV)-NL63 at 400 TCID_50_ per ml. After 96 h incubation at 32 °C in an atmosphere containing 5% CO_2_, cells were fixed and immunostained for HCoV-NL63 nucleoprotein (N) using specific antibodies. The rate of viral infection was analysed by flow cytometry and is presented as the percentage of cells that were infected. For comparisons by Student’s *t*-test, *Indicates *P* < 0.05. (**b**) LLC-Mk2 cells were transfected with capped and polyadenylated mRNA encoding active or catalytically inactive (m) APOBEC3 proteins. Following incubation for 24 h at 37 °C in an atmosphere containing 5% CO_2_, cells were lysed in RIPA buffer. APOBEC3 proteins and control GAPDH protein were detected by western blotting using specific antibodies. (**c**) 293T cell lysates enriched in the APOBEC3 protein A3C (lower panel) or green fluorescent protein (GFP) (upper panel) were incubated with *in vitro*-prepared RNA containing a GC-rich sequence for 2 h at 37 °C. The RNA was then reverse transcribed, amplified by PCR and sequenced. Experiments were performed at least three times, with two technical replicates. (**d**) Huh-7 cells were transfected with capped and polyadenylated mRNA encoding active A3F (lower panel) or GFP (upper panel). Following 24 h incubation at 37 °C in an atmosphere containing 5% CO_2_, cells were infected with the recombinant GC-rich HCoV-229E virus (ic229E-GC) at 400 TCID_50_ per ml. After four cycles of virus passage in A3F-positive or GFP-positive cells, viral RNA was isolated, reverse transcribed and sequenced across the GC-rich domain.

Because HCoV-NL63 infection resulted in upregulation of expression of A3A, A3C, A3D and A3F and A3C, A3F and A3H all inhibited HCoV-NL63 replication, we tested whether this inhibition was the result of the catalytic activity of the APOBECs. Plasmids encoding variants of A3C, A3F and A3H with Glu → Gln substitutions in the catalytic site were prepared^31^ and mRNAs encoding active or inactive proteins were transfected into LLC-Mk2 cells, which were infected with HCoV-NL63 and cultured prior to visualisation of viral proteins with specific antibodies. The percentages of HCoV-NL63-infected cells were measured by flow cytometry. Wild-type APOBEC3 proteins resulted in pronounced reductions in HCoV-NL63 infection rates, whereas catalytically inactive proteins produced moderate inhibition, with infection rates that were not significantly different from those in controls (Fig. 4a). Western blot analysis showed an even expression of all proteins, showing that the inhibitory effect did not result from unequal mRNA translation (Fig. 4b, Supplementary Figure 2). This level of inhibition of HCoV-NL63 replication by catalytically inactive proteins required transfection of 3 µg of mRNA, whereas for active proteins, inhibition was achieved with transfection of 1 µg of mRNA.

Next, we tested the activity of APOBEC3 proteins *in vitro*. A DNA template containing several possible sites for cytidine deamination (GC-rich domains) was used for *in vitro* transcription, producing RNA that was incubated with lysates from 293T cells overexpressing A3C protein or GFP. The RNA was then isolated, reverse transcribed, amplified by PCR and sequenced, revealing several C → C/T changes following incubation with A3C (but not GFP) cell lysate (Fig. 4c). However, hypermutation did not occur, and no pattern was identified in the nucleotide changes.

To examine the progressive accumulation of mutations, we performed a series of virus passages on cells expressing A3C, A3F or A3H. Notably, after the first passage, the inhibitory effect of the three APOBECs diminished, and no decline in virus yield was seen (data not shown). Cell-culture supernatants were collected after four passages of HCoV-NL63, and several regions of the viral RNA genome were sequenced. Point mutations (G → A or C → T) were identified in the genes encoding the 1ab polyprotein and spike protein in virus passaged in APOBEC3-expressing cells, but not in wild-type cells. These mutations were not specific to virus collected from cells expressing A3C, A3F or A3H, but were also identified in virus passaged in cells expressing A3A, ADE or A3G.

To identify whether APOBEC3 protein were capable of inducing hypermutation, we used a recombinant GC-rich HCoV-229E virus (ic229E-GC), into which we had introduced silent mutations (either A → G or T → C) in the A/T-rich region of nsp6, resulting in a potential ‘hot-spot’ for cytidine deamination. The virus was propagated and titrated to determine the 50% tissue-culture infective dose (TCID_50_) in naturally permissive Huh-7 cells. The effectiveness of mRNA transfection in Huh-7 cells was confirmed by detection of green fluorescence in cells transfected with GFP mRNA. Huh-7 cells were then transfected with mRNA encoding an APOBEC3 protein, infected with ic229E-GC at 400 TCID_50_ per ml and cultured for 5 days. Pronounced inhibition of viral infection occurred in cells expressing A3C, A3F or A3H (data not shown). After four passages of ic229E-GC, viral RNA was sequenced. No G → A or C → T mutations were identified within the GC-rich region in any of the samples. However, ic229E-GC passaged in cells expressing A3F protein showed considerable sequence heterogeneity, compared with passaged in GFP-positive cells, indicating a possible A3F deaminase activity (Fig. 4d).

### APOBEC3 proteins interact with the HCoV-NL63 nucleoprotein

**Figure 5:**
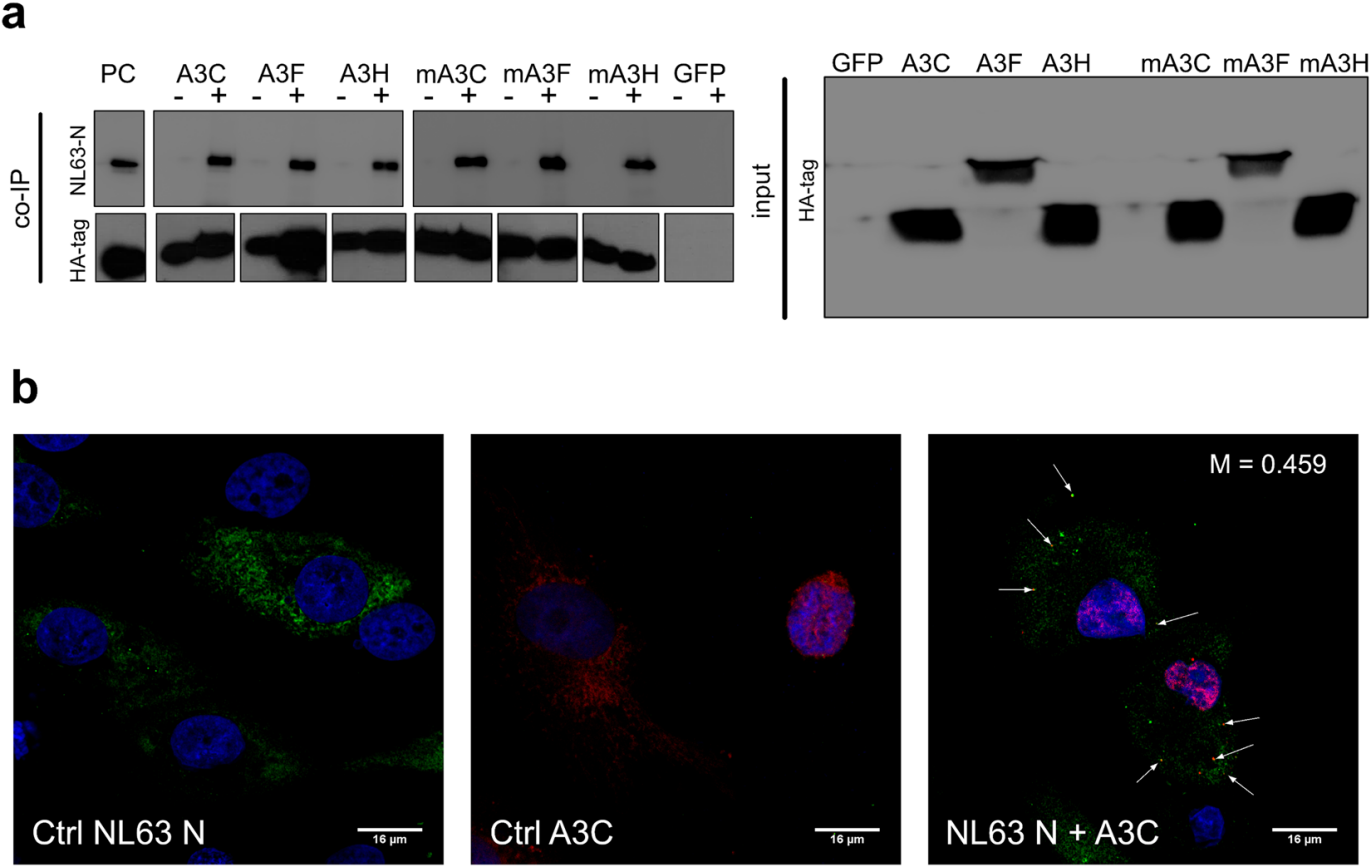
APOBEC3 proteins interact with human coronavirus (HCoV)-NL63 nucleoprotein (N). (**a**) 293T cell lysates enriched in active APOBEC3 proteins (A3C, A3F or A3H) or their catalytically inactive derivatives (mA3C, mA3F or mA3H) were incubated with purified HCoV-NL63 N (produced in *Escherichia coli*)^39^ for 1 h at 37 °C. Subsequently, agarose spheres coated with anti-HA antibodies were added, and proteins were co-precipitated using spin columns. Samples were separated by electrophoresis and transferred onto PVDF membrane, and HCoV-NL63 N and HA-tagged APOBEC3 proteins were detected with specific antibodies. PC = positive control protein. (**b**) LLC-Mk2 cells were transfected with capped and polyadenylated mRNA encoding HCoV-NL63 N (Ctrl NL63 N), A3C (Ctrl A3C) or both transcripts (NL63 N + A3C), and incubated for 72 h at 37 °C. Cells were then fixed and immunostained for A3C (red), HCoV-NL63 N (green) and nuclei (blue). Co-localisation of A3C with HCoV-NL63-N was determined by confocal microscopy and marked with arrows. M, Manders’ coefficient (A3C overlapping with the virus). A representative image is shown.

Our results demonstrated that, although cytidine deamination was involved in inhibition of coronavirus by APOBEC3 proteins, deaminase mutants retained some anti-coronaviral activity, suggesting that an additional mechanism may be involved in APOBEC3-mediated coronavirus restriction. We therefore analysed the interactions between APOBEC3 proteins (A3C, A3F and A3H) and HCoV-NL63 nucleocapsid protein (HCoV-NL63-N), which is the most abundant viral structural protein. Human 293T cells were transiently transfected with plasmid DNA constructs for expression of individual HA-tagged APOBEC3 proteins (or their catalytically inactive derivatives) or GFP. Cell lysates containing the expressed proteins were incubated with purified HCoV-NL63-N, and protein complexes were co-immunoprecipitated with anti-HA-tag resin. Western-blot analysis demonstrated co-immunoprecipitation of HCoV-NL63-N with A3C, A3F and A3H, whereas no HCoV-NL63-N could be detected in samples incubated with GFP lysate. Notably, the catalytically inactive derivatives of A3C, A3F and A3H also bound to HCoV-NL63-N protein (Fig. 5a, Supplementary Figure 3). On the other hand, we could not observe the same effect with other APOBEC3 proteins (A3A, A3D and A3G).

Co-localisation of A3C with HCoV-NL63-N was investigated by co-transfection of LLC-Mk2 cells with mRNAs encoding A3C and HCoV-NL63-N, followed by immunofluorescence staining and confocal microscopy. The analysis showed several clearly visible foci of co-localisation (Fig. 5b). Calculation of Manders’ coefficient gave a value of 0.459, indicating the existence of an interaction between the two proteins.

## Discussion

APOBEC3-mediated cytidine deamination has been described as an interim antiviral mechanism. Although APOBEC3 proteins were initially shown to primarily modify DNA templates, it has subsequently been demonstrated that they also use RNA as a substrate. The finding that APOBEC3 deaminases can mutate the HIV RNA genome first expanded their possible specificity^16^. Thereafter, it was shown that A3G impairs the replication of mumps, measles and respiratory syncytial viruses. This antiviral activity is not an effect of APOBEC3 enzymatic activity, but is thought to result from the interaction of A3G with viral transcripts^18^. A3A has been shown to act as a mRNA-editing factor in human monocytes and monocyte-derived macrophages^17^. However, APOBEC3-mediated deamination of viral RNA genomes has not previously been demonstrated for viruses that do not replicate through DNA intermediates. Here, we investigated the antiviral effects of APOBEC3 proteins on human coronaviruses. HCoV-NL63 was selected as the model pathogen because of its prominent bias in nucleotide composition (low GC content), suggesting the possible shaping of the genome by cytidine deamination.

We studied the interaction between HCoV and HAE cultures, as an *in vitro* model of the human airways, the primary entry site for human respiratory viruses. mRNA levels of genes encoding A3A, A3C and A3D were upregulated in HCoV-NL63-infected cultures. Upregulation of APOBEC3 protein expression was previously shown to be dependent on interferon alpha, and it is associated with non-cytolytic clearance of human hepatitis B virus in primary hepatocytes^32^. Cytoplasmic DNA triggers production of type I interferon, leading to massive upregulation of *APOBEC3A*^33,34^. Here, we investigated the effects of different APOBEC3 proteins, and found that expression of A3C, A3F or A3H limits coronavirus infection. Notably, some infectious progeny were produced in the presence of these proteins, and serial passaging of virus was possible. Surprisingly, if cells overexpressing A3C, A3F or A3H were infected with the progeny virions, no restriction of virus replication occurred. Possible explanations for this effect are that the first viral passage results in selection of viral subspecies that are not affected by APOBEC3 activities, or that viral passage activates a viral factor or mechanism that can counteract APOBEC3 activities.

Serial passage of HCoV in APOBEC3-expressing cells did not result in hypermutation in progeny viruses. It is possible that HCoV-NL63 has reached a maximum level of AT content, so that new C → T or G → A changes in the genome result in a drastic drop in viral infectivity. However, incubation of an RNA transcript containing potential deaminase hot-spots^17^ with A3C-enriched cell lysates did not result in hypermutation.

The lack of hypermutation might have resulted from limited accessibility of the template or instability of A3C in the lysate. We were not able to develop a HCoV-NL63 molecular clone containing GC-rich elements, most likely because of low stability of the reverse genetics system for this virus (data not shown). We therefore developed a HCoV-229E clone containing a GC-rich region^35,36^. Replication of this virus was inhibited in cells expressing A3C, A3F or A3H, but no mutations were observed in the modified region. The lack of observed hypermutation may result from proofreading and repair of the coronavirus RNA genome^37^, and suggests that inhibition of coronaviral replication by APOBEC3 proteins does not result from deamination of viral RNA.

It was previously shown that, for some RNA viruses, APOBEC-mediated inhibition is deamination-independent. In our system, catalytically inactive A3C, A3F and A3H proteins only had marginal inhibitory effect on virus replication. We suggest that APOBEC3 proteins may affect coronavirus replication by two mechanisms: first, by deamination of yet unidentified cellular targets or deamination of single sites in the coronaviral genome; and second, by direct, non-catalytic interaction with viral RNA and/or proteins. APOBEC proteins may interact with the coronaviral N protein, which is an essential structural protein for formation of the viral ribonucleocapsid, and which also has a role in virus replication^38–42^. Our results demonstrated that all APOBEC3 proteins with inhibitory activity bound to the N protein. The direct, editing-independent mechanism by which APOBEC3 proteins inhibit HCoV-NL63 replication is still to be elucidated, but it is possible that interaction with the N protein interferes with virus replication and progeny production.

In conclusion, our results show that three APOBEC3 proteins (A3C, A3F and A3H) inhibit coronaviral replication. The molecular mechanism of deaminase-dependent inhibition of coronavirus replication remains elusive and may depend on deamination of single sites in the coronaviral genome, or in a cellular target or targets. Passage of HCoV-NL63 in cells expressing APOBEC3 proteins results in virus that is resistant to APOBEC-mediated inhibition, possibly indicating the presence of an unidentified factor that counteracts APOBEC3 activity. The deaminase-independent component of viral inhibition may result from interaction between viral N protein and APOBEC3 proteins.

## Methods

### Cell culture

LLC-Mk2 cells (*Macaca mulatta* epithelial kidney cells; ATCC: CCL-7) were cultured in H/E MEM (Hanks’ MEM: Earle’s MEM, 2:1 ratio; Thermo Fisher Scientific, Poland) with 3% fetal bovine serum (heat-inactivated; Thermo Fisher Scientific, Poland) and antibiotics: penicillin (100 U/ml), streptomycin (100 μg/ml), and ciprofloxacin (5 μg/ml). Human hepatocellular carcinoma Huh-7 (kindly provided by dr Lia van der Hoek, UvA, The Netherlands) and 293T (ATCC: CRL-3216; kidney epithelial) cell lines were cultured in Dulbecco’s MEM (Thermo Fisher Scientific, Poland) with 10% fetal bovine serum (heat-inactivated; Thermo Fisher Scientific, Poland) and antibiotics: penicillin (100 U/ml), streptomycin (100 μg/ml), and ciprofloxacin (5 μg/ml). Cells were maintained at 37 °C under 5% CO_2_.

### Human airway epithelium (HAE) cultures

Human epithelial cells were isolated from conductive airways resected from transplant patients. The study was approved by the Bioethical Committee of the Medical University of Silesia in Katowice, Poland (approval no: KNW/0022/KB1/17/10 dated on 16.02.2010). A written informed consent was obtained from all patients. Tissues were procured at the Silesian Center for Heart Diseases, Zabrze, Poland. No organs/tissues were procured from prisoners. Cells were dislodged by protease treatment, and later mechanically detached from the connective tissue. Resulting primary cells were first cultured in selective media to proliferate. Further, cells were trypsinised and transferred onto permeable Transwell insert supports (ϕ = 6.5). Cell differentiation was stimulated by media additives and removal of media from the apical side. In such a manner cells were cultured for 6–8 weeks to form well-differentiated, pseudostratified mucociliary epithelium^43^. All experiments were performed in accordance with relevant guidelines and regulations.

For the virus infection, HAE cultures were washed thrice with 100 μl of 1 × PBS, following inoculation with HCoV-NL63 (isolate Amsterdam 1) or influenza A virus (IVA, strain H_3_N_2_) or mock (cell lysate). After 2 h incubation at 32° (for HCoV-NL63) or 37 °C (for IVA) unbound virions were removed by washing with 100 μl of 1 × PBS and HAE cultures were cultured at an air - liquid interphase until the end of the experiment.

### Virus preparation and titration

The stock of HCoV-NL63 (isolate Amsterdam 1) was prepared using LLC-Mk2 cells. Six days post-infection (p.i.) infected cells were lysed by freeze-thawing and the resulting supernatant was stored at −80 °C. Mock sample was prepared in the same manner, from non-infected cells. Virus yield was determined according to the method described by Reed and Muench^44^. Briefly, cells infected with serially diluted virus were incubated at 32 °C for 6 days and the appearance of cytopathic effect (CPE) was monitored.

### Isolation of nucleic acids and reverse transcription (RT)

Viral DNA/RNA Kit (A&A Biotechnology, Poland) was used for nucleic acid isolation from cell culture supernatants, according to the manufacturer’s instructions. Cellular RNA was isolated using Fenozol reagent (A&A Biotechnology, Poland), followed by DNase I treatment (Thermo Fisher Scientific, Poland). cDNA samples were prepared with a High Capacity cDNA Reverse Transcription Kit (Thermo Fisher Scientific, Poland), according to the manufacturer’s instructions.

### Preparation of GC-rich infectious clone 299E virus mutant

Recombinant HCoV-229E containing a GC-rich region was generated using the vaccinia virus-based reverse genetic system as described previously^36,45^. In short, vaccinia virus containing the full-length HCoV-229E cDNA in which an *Escherichia coli* guanine phosphoribosyltransferase (GPT) was inserted between HCoV-229E 10098 and 10930 nucleotides was used to recombine with a plasmid containing HCoV-229E 9398–11580 nucleotides with a number of silent mutations (GC-rich region)^46^. The resulting vaccinia virus was used to rescue the mutant HCoV-229E, as described previously^36,45^. Plasmid DNA, recombinant vaccinia virus and recombinant HCoV-229E identities were confirmed by sequencing.

### quantitative PCR (qPCR)

RNA yields were assessed using real-time PCR (7500 Fast Real-Time PCR; Life Technologies, Poland). HCoV-NL63 cDNA was amplified in a reaction mixture containing 1 × TaqMan Universal PCR Master Mix (Thermo Fisher Scientific, Poland), in the presence of FAM/TAMRA (6-carboxyfluorescein/6-carboxytetramethylrhodamine) probe (100 nM specific) and primers (450 nM each)^47^. Reaction was carried out according to the scheme: 2 min at 50 °C and 10 min at 92 °C, followed by 40 cycles of 15 sec at 92 °C and 1 min at 60 °C. Cellular cDNA was amplified in a reaction mixture containing 1 × SYBRGreen Ready PCR Master Mix (Sigma-Aldrich, Poland) in the presence of primers (500 nM each). TATA-box binding protein gene (TBP) was used as a household gene control and Rox was used as the reference dye. Primer sequences are provided in Table 1. Reaction was carried out according to the scheme: 10 min at 50 °C and 5 min at 95 °C, followed by 40 cycles of 30 sec at 95 °C, 30 sec at 56 °C and 45 sec at 75 °C. In order to assess the copy number for each gene, DNA standards were prepared, as described before^47^.

**Table 1:** Primers and probes used for a RT-qPCR.

Target	Primer	Primer sequence (5′–3′)
HCoV-NL63	Sense	CTG TGG AAA ACC TTT GGC ATC
	Antisense	CTG TGG AAA ACC TTT GGC ATC
	Probe	ATG TTA TTC AGT GCT TTG GTC CTC GTG AT
A3A	Sense	TGG TTC CTT CTT TGC AGT TGG ACC
	Antisense	GCA GCA TTT GCA GTG CCT CCT TAT
A3B	Sense	AGC ACA TGG GCT TTC TAT GCA ACG
	Antisense	AGG AGA TGA ACC AAG TGA CCC TGT
A3C	Sense	AAC GAA ACT TGG CTG TGC TTC ACC
	Antisense	AGA CAG TAT GTC GTC GCA GAA CCA
A3D	Sense	AGG CAG GAG GTG TAT TTC CGG TTT
	Antisense	GGT GCT CAG CCA AGA ATT TGG TCA
A3F	Sense	TCC GTG GAG ATC ATG GGC TAC AAA
	Antisense	TGC AGC TTG CTG TCC AGG AAT AGA
A3G	Sense	GCT GTG CCC AGG AAA TGG CTA AAT
	Antisense	ACA AAG GTG TCC CAG CAG TGC TTA
A3H	Sense	TGA CTT CAT CAA GGC TCA CGA CCA
	Antisense	GTC AGC AAA CTT TGG GAA GCC CAT
TBP	Sense	CCC ATG ACT CCC ATG ACC
	Antisense	TTT ACA ACC AAG ATT CAC TGT GG

### Flow cytometry

LLC-Mk2 cells cultured in 6-well plate format (TPP) were washed with sterile 1 × PBS, trypsinised, fixed with 4% formaldehyde, permeabilised with 0.1% Triton X-100 in 1 × PBS and incubated for 1 h with blocking buffer (10% BSA in 1 × PBS with 0.5% Tween 20).

To visualise HA-tagged APOBEC3 proteins after mRNA transfection, cells were incubated for 2 h at room temperature with mouse anti-HA-tag antibody (1 μg/ml, Antibodies-online, Germany) in 1 × PBS with 3% BSA and 0.5% Tween 20, followed by 1 h incubation with Alexa Fluor 488-labeled goat anti-mouse antibody (2.5 μg/ml, Molecular Probes, Poland).

To evaluate HCoV-NL63 infection using flow cytometry, cells were incubated for 2 h at room temperature with mouse anti-HCoV-NL63-N antibody (1 μg/ml, Ingenansa, Spain) in 1 × PBS with 3% BSA and 0.5% Tween 20, followed by 1 h incubation with Alexa Fluor 488-labeled goat anti-mouse antibody (2.5 μg/ml, Molecular Probes, Poland). Cells were then washed, re-suspended in 1 × PBS and analysed with FACS Calibur (Becton Dickinson, USA) using Cell Quest software.

### APOBEC3 expression constructs and site-directed mutagenesis

Sequence verified, C-terminal HA-tagged pTracer CMV2 plasmids (Thermo Fisher Scientific, Poland) encoding human A3A (GeneBank accession number (GB) NM_145699), A3C (GB: NM_014508), A3DE (GB: NM_152426), A3F (GB: NM_145298), APOBEC3G (GB: NM_021822) and A3H (GB: NM_181773) were prepared. Catalytic mutants of A3C (A3C_E68Q = mA3C), A3F (A3F_E251Q = mA3F) and A3H (A3H_E56Q = mA3H) were prepared using Quick Change mutagenesis with Phusion polymerase (Thermo Fisher Scientific, Poland)^48^. Sequences of oligonucleotides used are listed in Table 2.

**Table 2:** Primers used for generation of expression constructs and deaminase mutants.

Target	Primer	Primer sequence (5′–3′)
**Oligonucleotides used for plasmids construction**		
A3A	Sense	ACG CGA ATT CCA CCA TGG AAG CCA GCC CAG CAT CCG G
	Antisense	GAC TGC GGC CGC CTA TCA AGC GTA ATC TGG AAC ATC GTA TGG GTA GTT TCC CTG ATT CTG GAG AA
A3C	Sense	ACG CGA ATT CCA CCA TGA ATC CAC AGA TCA GAA ACC CGA TG
	Antisense	GAC TGC GGC CGC CTA TCA AGC GTA ATC TGG AAC ATC GTA TGG GTA CTG GAG ACT CTC CCG TAG CC
A3D	Sense	ACG CGA ATT CCA CCA TGA ATC CAC AGA TCA GAA ATC C
	Antisense	GAC TGC GGC CGC CTA TCA AGC GTA ATC TGG AAC ATC GTA TGG GTA CTG GAG AAT CTC CCG TAG CC
A3F	Sense	ACG CGG TAC CCA CCA TGA AGC CTC ACT TCA GAA ACA C
	Antisense	GAC TGC GGC CGC CTA TCA AGC GTA ATC TGG AAC ATC GTA TGG GTA CTC GAG AAT CTC CTG CAG CT
A3G	Sense	ACG CGG TAC CCA CCA TGA AGC CTC ACT TCA GAA ACA C
	Antisense	GAC TGC GGC CGC CTA TCA AGCG TAA TCT GGA ACA TCG TAT GGG TAG TTT TCC TGA TTC TGG AGA A
A3H	Sense	ACG CGA ATT CCA CCA TGG CTC TGT TAA CAG CCG AAA C
	Antisense	GAC TGC GGC CGC CTA TCA AGC GTA ATC TGG AAC ATC GTA TGG GTA GAC CTC AGC ATC ACAC AAT A
**Oligonucleotides used for Quick Change mutagenesis**		
mA3C	Sense	GTC ATG CAC AAA GGT GCT TCC TCT CTT G
	Antisense	GCA CCT TTG TGC ATG ACA ATG GGT CTC
mA3F	Sense	GTC ATG CAC AAA GGT GCT TCC TCT CTT G
	Antisense	GCA CCT TTG TGC ATG ACA ATG GGT CTC
mA3H	Sense	GCC ATG CAC AAA TTT GCT TTA TTA ACG
	Antisense	GCA AAT TTG TGC ATG GCA CTT TTT CTT G

### Design of “hot-spot” RNA

Plasmid construct containing several GC-rich domains was ordered at GeneArt Gene Synthesis (Thermo Fisher Scientific, Germany). The region was sequenced with primers 5′-CTG TGG AAA ACC TTT GGC ATC-3′ and 5′-CTG TGG AAA ACC TTT GGC ATC-3′. The plasmid was amplified in *E*. *coli* TOP10 strain and further used as a template in *in vitro* transcription reaction using T7 promoter, as described below.

### Preparation of mRNA molecules for transfection

APOBEC3 plasmids described above were used as templates for amplification of APOBEC3 genes with forward primer containing the T7 promoter region (5′-TCG GCC TCG TAG GCC TAA TAC GAC TCA CTA TAG GGA GAC TGA GAG AAC CCA CTG CTT AC-3′) and specific reverse primer (listed in Table 2, 500 nM each). Reaction was carried out with Phusion polymerase (Thermo Fisher Scientific, Poland), according to the manufacturer’s instructions. DNA templates were purified using GeneJET PCR Purification Kit (Thermo Fisher Scientific, Poland) and further used for *in vitro* transcription with T7 RiboMAX Express Large Scale RNA Production System and Ribo m7G Cap Analog (Promega, Poland). Resulting RNA was precipitated with LiCl, purified with 70% ethanol and polyadenylated using Poly(A) Tailing Kit (Thermo Fisher Scientific, Poland), according to the manufacturer’s instructions. RNA was then precipitated with lithium chloride (Thermo Fisher Scientific, Poland) and its concentration was assessed using a spectrophotometer.

### Transfection of plasmid DNA and mRNA

293T cells were seeded on 10 cm^2^ dishes, cultured for 24 h at 37 °C with 5% CO_2_ and transfected with 8 µg of plasmid per dish using polyethylenimine (Sigma-Aldrich, Poland). LLC-Mk2 and Huh-7 cells were seeded on 6-wells plates (TPP, Switzerland), cultured for 24 h at 37 °C with 5% CO_2_ and transfected with 3 µg mRNA per well (transcripts encoding APOBEC3 proteins or control GFP) using Trans*IT* mRNA Transfection Reagent (Mirus, USA). Efficiency of transfection was assessed 24 h post-transfection in GFP-transfected cells using EVOS fluorescent microscope (Thermo Fisher Scientific, Poland).

### Western blot analysis

Cells were trypsinised, centrifuged and resuspended in RIPA buffer (50 mM Tris, 150 mM NaCl, 1% Nonidet P-40, 0.5% sodium deoxycholate, 0.1% SDS, pH 7.5) followed by lysis in RIPA buffer for 30 min on ice. Subsequently, samples were centrifuged (10 min at 12,000 × g) and the pelleted cell debris was discarded. Resulting supernatants were mixed with sample buffer (0.5 M Tris pH 6.8, 10% SDS, 50 mg/ml DTT), boiled for 5 min, cooled on ice, and separated on 10% polyacrylamide gels alongside dual color Page Ruler Pre-stained Protein size markers (Thermo Fisher Scientific, Poland). The separated proteins were then transferred onto a Westran S PVDF membrane (GE Healthcare, Poland) by wet blotting (Bio-Rad, Poland) for 1 h, 100 Volts in transfer buffer: 25 mM Tris, 192 mM glycine, 20% methanol at 4 °C. The membranes were then blocked by overnight incubation at 4 °C in TBS-Tween (0.1%) buffer supplemented with 5% skimmed milk (BioShop, Canada). A rabbit anti-HA Taq antibody (1 μg/ml; Antibodies-online, USA) and horseradish peroxidase-labeled goat anti-rabbit IgG (0.35 μg/ml; Sigma-Aldrich, Poland) were used to detect the HA-tagged APOBEC3 proteins in cell lysates. Mouse anti-HCoV-NL63-N protein antibody (500 ng/ml; Ingenansa, Spain) and horseradish peroxidase-labeled rabbit anti-mouse IgG (65 ng/ml; Dako, Denmark) were used to detect the HCoV-NL63 nucleocapsid protein. A rabbit anti-GAPDH antibody (1:5000 dilution; Cell Signalling Technology, Poland) and horseradish peroxidase-labeled goat anti-rabbit IgG (0.35 μg/ml; Sigma-Aldrich, Poland) were used to detect the GAPDH in cell lysates. All antibodies were diluted in 1% skimmed milk/TBS-Tween (0.1%). The signal was developed using the Immobilon Western Chemiluminescent HRP Substrate (Millipore, USA) and visualised by exposing the membrane to an X-ray film (Kodak, Poland).

### Nucleocapsid protein binding analysis

APOBEC3-expressing and control 293T cells were harvested 3 days post transfection in deaminase activity buffer, containing 0.2% Surfact-Amps NP-40 (Sigma-Aldrich, Poland), 30 mM 4-(2-hydroxyethyl)-1-piperazine-ethane-sulfonic acid (HEPES; pH 7.5), 100 mM KCl, 25 mM NaCl, 1.5 mM MgCl_2_, 1 mM DTT, 1 × Halt protease and phosphatase inhibitor cocktail, and 10% glycerol^17^. Samples were centrifuged (10 min at 12,000 × g), pelleted cell debris was discarded and resulting supernatants were stored at −80 °C. HCoV-NL63 N protein was prepared as described previously^39^. Cell lysates were incubated for 1 h at 37 °C with the purified N protein in 1 × activity buffer, containing 100 mM KCl, 10 mM HEPES (pH 7.4), 100 µM ZnCl_2_, 1 mM DTT and 1 mM EDTA^17^. Subsequently, 15 µl of agarose spheres coated with anti-HA antibodies (Pierce, Poland) was added and samples were loaded onto co-immunoprecipitation columns. After overnight incubation at 4 °C on a rotary shaker, proteins were eluted, according to the manufacturer’s instructions.

### Analysis of HCoV-NL63 co-localisation with APOBEC3C

LLC-Mk2 cells were seeded on 6-wells plates and cultured for 24 h at 37 °C with 5% CO_2_. 1.5 μg of HCoV-NL63 N mRNA and 1.5 μg of A3C mRNA was co-transfected using the mRNA-In reagent (AMS Biotechnology, United Kingdom). After 72 h incubation at 37 °C with 5% CO_2_, cells were fixed with 4% formaldehyde, permeabilised with 0.1% Triton X-100 in 1 × PBS and incubated overnight at 4 °C with 10% BSA in 1 × PBS with 0.5% Tween 20. To visualise HCoV-NL63 N molecules, cells were incubated for 2 h at room temperature with rabbit anti-HCoV-NL63-N serum (1:100 dilution, kindly provided by dr Lia van der Hoek, UvA, The Netherlands) in 1 × PBS with 3% BSA and 0.5% Tween 20, followed by 1 h incubation with Atto 488-labeled goat anti-rabbit IgG (5 μg/ml; Sigma-Aldrich, Poland). To detect HA-tagged A3C protein cells were incubated for 2 h at room temperature with mouse Alexa Fluor 647-labeled anti-HA-tag antibody (10 μg/ml; Thermo Fisher Scientific; Poland) in 1 × PBS with 3% BSA and 0.5% Tween 20. Nuclear DNA was stained with DAPI (0.1 μg/ml; Sigma-Aldrich, Poland). Immunostained cultures were mounted on glass slides with ProLong Gold antifade medium (Thermo Fisher Scientific; Poland). Fluorescent images were acquired under a Zeiss LSM 710 confocal microscope (Carl Zeiss Microscopy GmbH). Images were acquired using ZEN 2012 SP1 software (Carl Zeiss Microscopy GmbH) and processed using ImageJ 1.47 v (National Institutes of Health, Bethesda, Maryland, USA).

### Statistical analysis

All the experiments were performed in triplicate and the results are presented as mean ± SD. Student’s t-test was used to determine significance of the obtained results. *P* values < 0.05 were considered significant.

## Electronic supplementary material

Supplementary figures 1, 2 and 3

## References

[1] Jarmuz A (2002). An anthropoid-specific locus of orphan C to U RNA-editing enzymes on chromosome 22. Genomics.

[2] Esnault C, Millet J, Schwartz O, Heidmann T (2006). Dual inhibitory effects of APOBEC family proteins on retrotransposition of mammalian endogenous retroviruses. Nucleic Acids Res.

[3] Madani N, Kabat D (1998). An endogenous inhibitor of human immunodeficiency virus in human lymphocytes is overcome by the viral Vif protein. J Virol.

[4] Sheehy AM, Gaddis NC, Choi JD, Malim MH (2002). Isolation of a human gene that inhibits HIV-1 infection and is suppressed by the viral Vif protein. Nature.

[5] Dang Y, Wang X, Esselman WJ, Zheng YH (2006). Identification of APOBEC3DE as another antiretroviral factor from the human APOBEC family. J Virol.

[6] Hultquist JF (2011). Human and rhesus APOBEC3D, APOBEC3F, APOBEC3G, and APOBEC3H demonstrate a conserved capacity to restrict Vif-deficient HIV-1. J Virol.

[7] Zielonka J (2009). Restriction of equine infectious anemia virus by equine APOBEC3 cytidine deaminases. J Virol.

[8] Bishop KN (2004). Cytidine deamination of retroviral DNA by diverse APOBEC proteins. Curr Biol.

[9] Kobayashi M (2004). APOBEC3G targets specific virus species. J Virol.

[10] Löchelt M (2005). The antiretroviral activity of APOBEC3 is inhibited by the foamy virus accessory Bet protein. Proc Natl Acad Sci USA.

[11] Delebecque F (2006). Restriction of foamy viruses by APOBEC cytidine deaminases. J Virol.

[12] Noguchi C (2005). G to A hypermutation of hepatitis B virus. Hepatology.

[13] Suspène R (2005). Extensive editing of both hepatitis B virus DNA strands by APOBEC3 cytidine deaminases *in vitro* and *in vivo*. Proc Natl Acad Sci USA.

[14] Chen H (2006). APOBEC3A is a potent inhibitor of adeno-associated virus and retrotransposons. Curr Biol.

[15] Yang B, Chen K, Zhang C, Huang S, Zhang H (2007). Virion-associated uracil DNA glycosylase-2 and apurinic/apyrimidinic endonuclease are involved in the degradation of APOBEC3G-edited nascent HIV-1 DNA. J Biol Chem.

[16] Bishop KN, Holmes RK, Sheehy AM, Malim MH (2004). APOBEC-mediated editing of viral RNA. Science.

[17] Sharma S (2015). APOBEC3A cytidine deaminase induces RNA editing in monocytes and macrophages. Nat Commun.

[18] Fehrholz M (2012). The innate antiviral factor APOBEC3G targets replication of measles, mumps and respiratory syncytial viruses. J Gen Virol.

[19] van der Hoek L (2005). Croup is associated with the novel coronavirus NL63. PLoS Med.

[20] Pyrc K, Berkhout B, van der Hoek L (2007). The novel human coronaviruses NL63 and HKU1. J Virol.

[21] Pyrc K (2006). Mosaic structure of human coronavirus NL63, one thousand years of evolution. J Mol Biol.

[22] Woo PC (2005). Characterization and complete genome sequence of a novel coronavirus, coronavirus HKU1, from patients with pneumonia. J Virol.

[23] Peiris JS, Yuen KY, Osterhaus AD, Stöhr K (2003). The severe acute respiratory syndrome. N Engl J Med.

[24] Stadler K (2003). SARS–beginning to understand a new virus. Nat Rev Microbiol.

[25] de Groot RJ (2013). Middle East respiratory syndrome coronavirus (MERS-CoV): announcement of the Coronavirus Study Group. J Virol.

[26] Zaki AM, van Boheemen S, Bestebroer TM, Osterhaus AD, Fouchier RA (2012). Isolation of a novel coronavirus from a man with pneumonia in Saudi Arabia. N Engl J Med.

[27] van der Hoek L (2004). Identification of a new human coronavirus. Nat Med.

[28] Pyrc K, Jebbink MF, Berkhout B, van der Hoek L (2004). Genome structure and transcriptional regulation of human coronavirus NL63. Virol J.

[29] Woo PC, Wong BH, Huang Y, Lau SK, Yuen KY (2007). Cytosine deamination and selection of CpG suppressed clones are the two major independent biological forces that shape codon usage bias in coronaviruses. Virology.

[30] Pauli EK (2009). High level expression of the anti-retroviral protein APOBEC3G is induced by influenza A virus but does not confer antiviral activity. Retrovirology.

[31] Albin JS, Brown WL, Harris RS (2014). Catalytic activity of APOBEC3F is required for efficient restriction of Vif-deficient human immunodeficiency virus. Virology.

[32] Bonvin M (2006). Interferon-inducible expression of APOBEC3 editing enzymes in human hepatocytes and inhibition of hepatitis B virus replication. Hepatology.

[33] Suspène R (2017). Self-cytoplasmic DNA upregulates the mutator enzyme APOBEC3A leading to chromosomal DNA damage. Nucleic Acids Res.

[34] Clementz MA (2010). Deubiquitinating and interferon antagonism activities of coronavirus papain-like proteases. J Virol.

[35] Donaldson EF (2008). Systematic assembly of a full-length infectious clone of human coronavirus NL63. J Virol.

[36] Thiel V, Herold J, Schelle B, Siddell SG (2001). Infectious RNA transcribed *in vitro* from a cDNA copy of the human coronavirus genome cloned in vaccinia virus. J Gen Virol.

[37] Smith EC, Denison MR (2013). Coronaviruses as DNA wannabes: a new model for the regulation of RNA virus replication fidelity. PLoS Pathog.

[38] Fields, B. N., Knipe, D. M. & Howley, P. M. *Fields virolog*y. 6th edn, (Wolters Kluwer Health/Lippincott Williams & Wilkins, 2013).

[39] Zuwała K (2015). The nucleocapsid protein of human coronavirus NL63. PLoS One.

[40] Hiscox JA (2001). The coronavirus infectious bronchitis virus nucleoprotein localizes to the nucleolus. J Virol.

[41] Hurst KR, Koetzner CA, Masters PS (2009). Identification of *in vivo*-interacting domains of the murine coronavirus nucleocapsid protein. J Virol.

[42] Wang SM, Wang CT (2009). APOBEC3G cytidine deaminase association with coronavirus nucleocapsid protein. Virology.

[43] Milewska A (2013). Novel polymeric inhibitors of HCoV-NL63. Antiviral Res.

[44] Reed, L. & Muench, H. A simple method of estimating fifty per cent endpoints. *Am. J. Epidemiol* **27**, 493–497 (1938).

[45] Eriksson KK, Makia D, Thiel V (2008). Generation of recombinant coronaviruses using vaccinia virus as the cloning vector and stable cell lines containing coronaviral replicon RNAs. Methods Mol Biol.

[46] Lundin A (2014). Targeting membrane-bound viral RNA synthesis reveals potent inhibition of diverse coronaviruses including the middle East respiratory syndrome virus. PLoS Pathog.

[47] Milewska A (2016). HTCC: Broad Range Inhibitor of Coronavirus Entry. PLoS One.

[48] Bok JW, Keller NP (2012). Fast and easy method for construction of plasmid vectors using modified quick-change mutagenesis. Methods Mol Biol.

