# Supplementary material for: APOBEC3-mediated restriction of RNA virus replication
Source: Sci Rep. 2018 Apr 13;8:5960. doi: 10.1038/s41598-018-24448-2 (PMC5899082; doi:10.1038/s41598-018-24448-2)
Supplement: Supplementary file 1 — Supplementary figures 1, 2 and 3 [file 41598_2018_24448_MOESM1_ESM.pdf]

Apobec3-mediated restriction of RNA virus replication

Aleksandra Milewska[a,b,*], Eveline Kindler[c,d], Philip Vkovski[c,d,e], Slawomir Zeglen[f,g], Marek Ochman[h], Volker Thiel[c,d], Zenon Rajfur[i], Krzysztof Pyrc[a,b,*]

[a] Microbiology Department, Faculty of Biochemistry, Biophysics and Biotechnology, Jagiellonian University, Gronostajowa 7, 30-387 Krakow, Poland.
[b] Virogenetics Laboratory of Virology, Malopolska Centre of Biotechnology, Jagiellonian University, Gronostajowa 7a, 30–387 Krakow, Poland.
[c] Institute for Virology and Immunology, Bern and Mittelhäusern, Switzerland.
[d] Department of Infectious Diseases and Pathobiology, Vetsuisse Faculty, University of Bern, Länggassstrasse 122, Bern, Switzerland.
[e] Graduate School for Cellular and Biomedical Sciences, University of Bern, Switzerland.
[f] Department of Cardiac Surgery and Transplantology, Silesian Center for Heart Diseases, Marii Curie-Skłodowskiej 9, 41-800 Zabrze, Poland.
g Head of Histology Department, Medical Department, University of Opole
[h] Department of Pharmacology, School of Medicine with the Division of Dentistry in Zabrze, Medical University of Silesia in Katowice
[i] Institute of Physics, Faculty of Physics, Astronomy and Applied Computer Sciences, Jagiellonian University, Lojasiewicza 11, 30-348 Krakow, Poland.

[*] **Corresponding authors**

* Correspondence should be addressed to **Aleksandra Milewska** (aleksandra.milewska@uj.edu.pl) or **Krzysztof Pyrc (**k.a.pyrc@uj.edu.pl), Microbiology Department, Faculty of Biochemistry, Biophysics and Biotechnology, Jagiellonian University, Gronostajowa 7, 30-387 Krakow, Poland; Phone number: +48 12 664 61 21; Fax: +48 12 664 69 02.

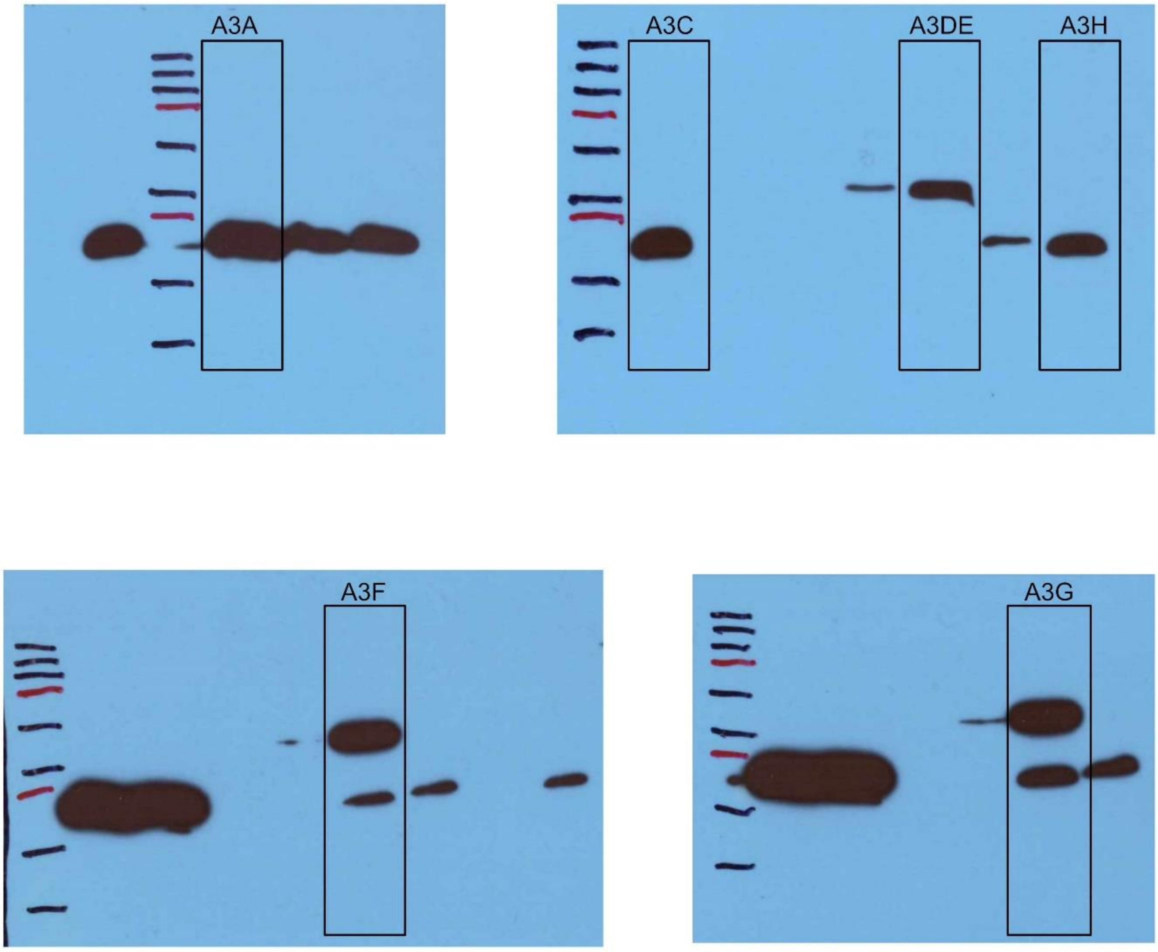

Supplementary Figure 1. Full-size Western blots from Figure 2a.

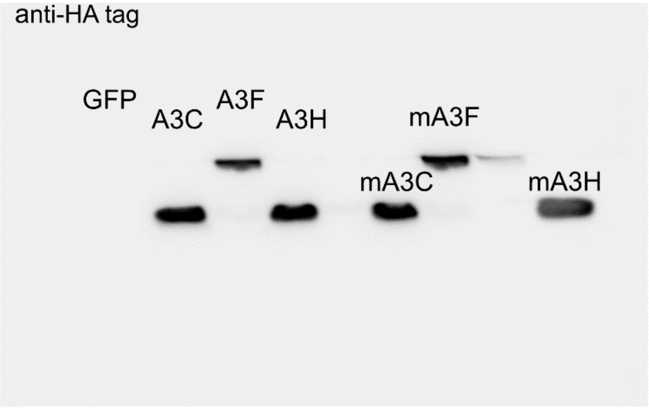

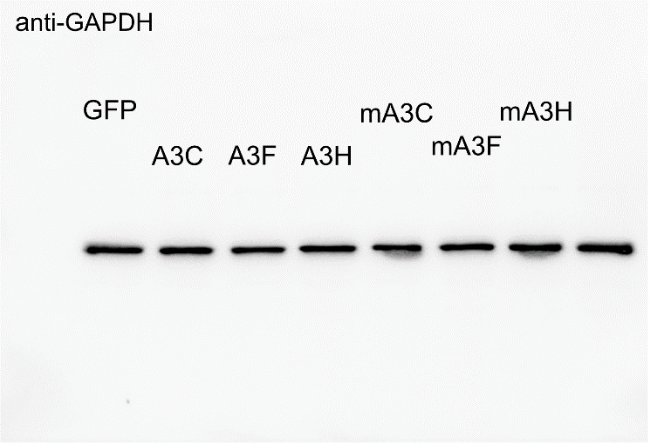

Supplementary Figure 2. Full-size Western blots from Figure 4b.

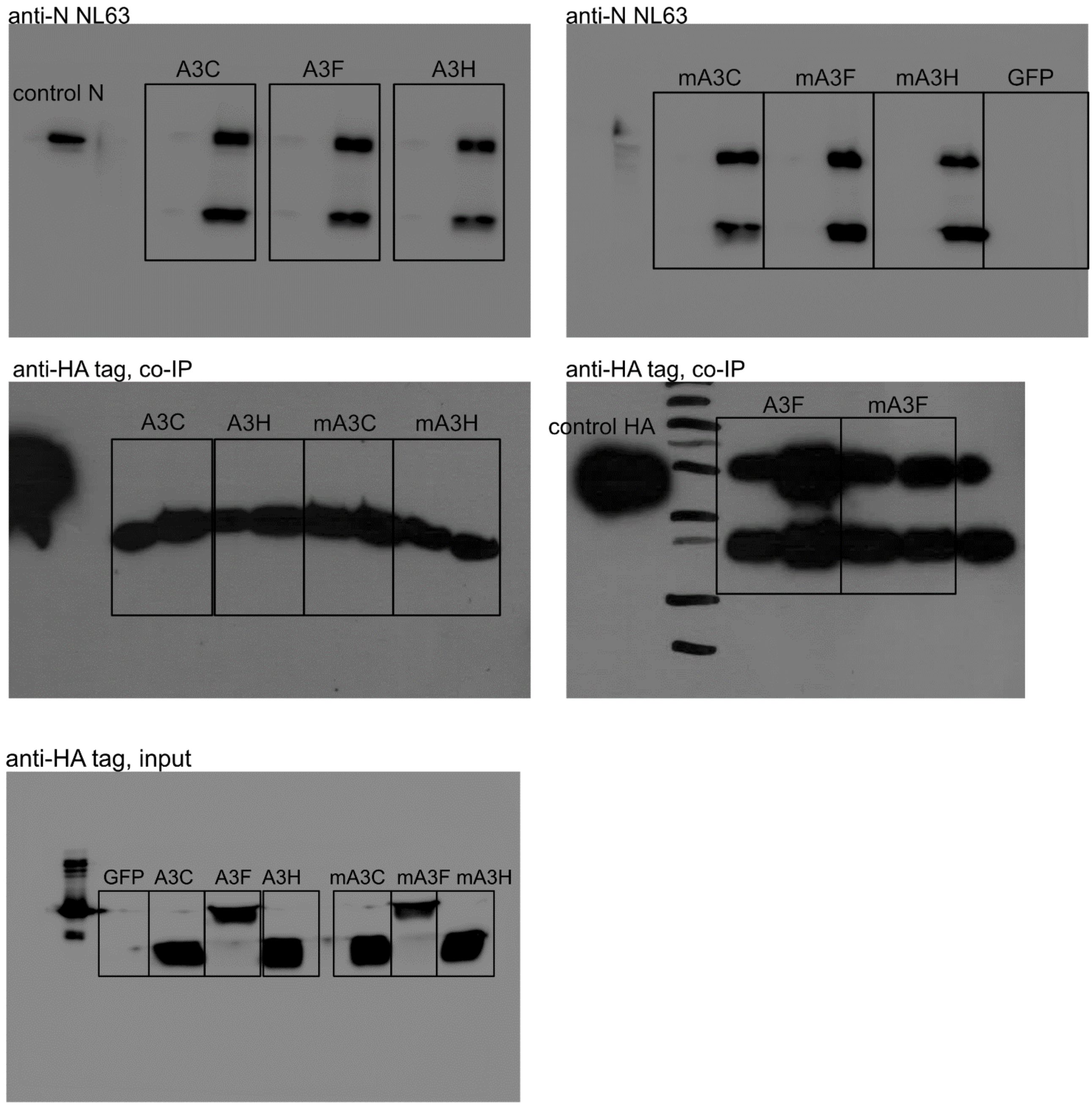

Supplementary Figure 3. Full-size Western blots from Figure 5a.
